# Supplementary material for: Alpha/beta power decreases track the fidelity of stimulus-specific information
Source: eLife. 2019 Nov 29;8:e49562. doi: 10.7554/eLife.49562 (PMC6904219; doi:10.7554/eLife.49562)
Supplement: Supplementary file 1. [file elife-49562-supp1.docx]

**Supplementary Table 1.** fMRI cluster-based statistics with standard thresholding compared to more conservative thresholding.

| **Analysis** | **p<.001, min 10 voxels** | | | | | **p<.0001, min 50 voxels** | | | |
| --- | --- | --- | --- | --- | --- | --- | --- | --- | --- |
|  | *Cluster* | *p_FWE_* | *k* | *MNI* | *Cluster* | | *p_FWE_* | *k* | *MNI* |
| **Visual Perception RSA** | Occip. | p<.001 | 9911 | [-30,-67,-2] | Occip. | | p<.001 | 6072 | [-30,-67,-2] |
|  | Temp. | p=.003 | 64 | [-48,-1,18] | *No cluster formed* | | | | |
|  | Cingulate | p<.001 | 113 | [12,-16,50] | *No cluster formed* | | | | |
| **Audio Perception RSA** | L. Temp. | p<.001 | 698 | [-57,-37,10] | *No cluster formed* | | | | |
|  | R. Temp. | p<.001 | 859 | [60,-25,10] | R. Temp. | | p<.001 | 425 | [60,-25,10] |
| **Retrieval RSA** | L. Fusi. | p<.001 | 472 | [-45,-37,-6] | L. Fusi. | | p<.001 | 214 | [-45,-37,-6] |
|  | R. Fusi. | p<.001 | 270 | [27,-52,-10] | R. Fusi. | | p<.001 | 55 | [27,-52,-10] |
| **Encoding Vis > Aud.** | Occip. | p<.001 | 975 | [42,-70,10] | R. Occip. | | p<.001 | 367 | [42,-70,10] |
|  |  |  |  |  | L. Occip. | | p<.001 | 322 | [-24,-88,10] |
|  | L. Temp. | p=.008 | 67 | [-48,2,-10] | *No cluster formed* | | | | |
|  | R.Temp. | p=.005 | 72 | [48,5,-14] | *No cluster formed* | | | | |
| **Retrieval Vis. > Aud.** | L. Fusi. | p=.001 | 89 | [-30,-46,-6] | *No cluster formed* | | | | |
|  | R Fusi. | p=.001 | 99 | [21,-37,-14] | *No cluster formed* | | | | |
| **Retrieval Hit > Miss** | Occip. | p<.001 | 1178 | [12,-52,-14] | Occip. | | p<.001 | 543 | [12,-52,-14] |
|  | Limbic | p<.001 | 1447 | [-21,-16,2] | L. Limbic | | p<.001 | 360 | [-21,-16,2] |
|  |  |  |  |  | R. Limbic | | p<.001 | 54 | [27,5,10] |
| **Power * BOLD Hit > Miss** | Occip. | p<.001 | 5183 | [-6,-76,14] | Occip. | | P<.001 | 715 | [-6,-76,14] |
|  | Parietal | p<.001 | 139 | [39,-40,38] | Parietal | | p<.001 | 51 | [39,-40, 38] |
